# Role of Gut Microbiota in Early Infant Development

**DOI:** 10.4137/cmped.s2008

**Published:** 2009-03-04

**Authors:** R Wall, R.P Ross, C.A Ryan, S Hussey, B Murphy, G.F Fitzgerald, C Stanton

**Affiliations:** 1 Alimentary Pharmabiotic Centre (APC), Co. Cork, Ireland.; 2 Teagasc, Moorepark Food Research Centre, Fermoy, Co. Cork, Ireland.; 3 University College Cork, National University of Ireland, Ireland.; 4 Department of Paediatrics and Child Health, University College Cork, Ireland.

**Keywords:** infant, gut microbiota, colonization, infant health, probiotics, prebiotics

## Abstract

Early colonization of the infant gastrointestinal tract is crucial for the overall health of the infant, and establishment and maintenance of non-pathogenic intestinal microbiota may reduce several neonatal inflammatory conditions. Much effort has therefore been devoted to manipulation of the composition of the microbiota through 1) the role of early infant nutrition, particularly breast milk, and supplementation of infant formula with prebiotics that positively influence the enteric microbiota by selectively promoting growth of beneficial bacteria and 2) oral administration of probiotic bacteria which when administered in adequate amounts confer a health benefit on the host. While the complex microbiota of the adult is difficult to change in the long-term, there is greater impact of the diet on infant microbiota as this is not as stable as in adults. Decreasing excessive use of antibiotics and increasing the use of pre- and probiotics have shown to be beneficial in the prevention of several important infant diseases such as necrotizing enterocolitis and atopic eczema as well as improvement of short and long-term health. This review addresses how the composition of the gut microbiota becomes established in early life, its relevance to infant health, and dietary means by which it can be manipulated.

## Introduction

The neonatal period is crucial for intestinal colonization, and the processes involved in the establishment of microbial populations are complex and involve both microbial succession as well as interactions between the infant and the microbes in the different regions of the gut.

However, there are conflicting reports in the literature regarding the composition of the neonatal gastrointestinal microbiota and the factors that shape it. The gastrointestinal tract (GIT) of the fetus is sterile but becomes rapidly colonized in the early days of life, influenced by factors such as the mode of delivery, the maternal microbiota, milk source and the surrounding environment.^1^–^5^ Changes in the colonization pattern occur up to two years of age, when the microbiota stabilizes and resembles that of adulthood. When established, the adult gut contains trillions of microbes with a collective genome that outnumbers the human genome by up to 1000-fold.^6^ Emerging evidence points to a dynamic and generally favorable, symbiotic relationship between humans and their enteric microbiota. The diverse bacterial species within this ecosystem each contain a battery of enzymes capable of performing a myriad of different functions, ranging from transformation of substances present in the gut (to less or more toxic compounds), the production of antimicrobial substances active against pathogenic bacteria and stimulation of the immune system.^7^,^8^ Moreover, it has been demonstrated that some commensals within the enteric microbiota are able to produce a range of bioactive fatty acids and metabolites such as conjugated linoleic acid (CLA), short chain fatty acids (SCFA) and gamma-amino butyric acid (GABA) which have shown great potential in the treatment of lifestyle diseases including cancer, obesity and cardiovascular disease.^9^–^13^ While species of bacteria are found in the acidic conditions of the stomach, the bacterial density progressively increases distally in the intestine.^14^ Anaerobic and aerobic genera of bacteria inhabit the GIT,^14^,^15^ though the majority are strict anaerobes^16^ with *Bifidobacterium*, *Clostridium*, *Bacteroides*, *Lactobacillus* and *Eubacterium* being among the most commonly found enteric bacteria.^15^,^16^

Since colonization with a non-pathogenic microbiota is essential to infant health and probably also has an effect on overall health status in later life, it is important to understand how the composition of this gut microbial ecosystem is established. Moreover, given the importance of the establishment of a healthy GIT in early life, different strategies have evolved to manipulate the microbiota particularly by using prebiotic supplementation and probiotic administration.^17^,^18^

## The Development of the Gut Microbiota in the Infant

The predominant sources of microbes for the initial colonization of the GIT following birth are the maternal microbiota, especially during vaginal delivery, and the infant’s diet (breast versus formula feeding). Other factors that influence the composition of the enteric microbiota of infants are the environment during birth, gestational age, hygiene measures and antibiotic treatment.^3^ Microbes have also been detected in amniotic fluid and placenta from mothers and in the umbilical cord blood of healthy neonates,^19^ suggesting that these bacteria may also be part of the first colonizers in the GIT of the newborn.

Mode of delivery is a key factor that shapes the developing infant microbiota^3^–^5^ and in this respect, infants delivered by Caesarean section have been reported to harbor an enteric microbiota that differs from vaginally delivered infants, both in the timing of colonization and in composition.^3^–^5^,^20^,^21^ Vaginally born infants are initially colonized by fecal and vaginal bacteria from the mother,^21^–^24^ whereas infants born via Caesarean section are exposed initially to bacteria originating from the hospital environment and health-care workers.^23^,^21^,^25^ It has been reported that approximately one quarter of infants acquire vaginal lactobacilli from their mothers at birth.^24^ The microbiota of infants born by Caesarean section is characterized by lower numbers of strict anaerobes such as *Bacteroides fragilis* and bifidobacteria compared to vaginally delivered infants.^3^,^20^,^21^,^26^ The colonization of these infants is also often delayed, and it may take up to one month before similar numbers of bacteria are present compared with vaginally delivered infants.^21^ Moreover, the prevalence and numbers of *Clostridium difficile* and *Escherichia coli* are generally higher in infants born by Caesarean section.^3^ Compared with vaginally born infants, the median counts of *B. fragilis* group bacteria and *C. difficile* were shown to be ∼100-fold lower and ∼100-fold higher, respectively, for infants born via Caesarean section.^3^ It is difficult to assess the influence of the delay in intestinal colonization following Caesarean section on the development of the gut-associated immune system. However, the balance between *Bifidobacterium* and *Clostridium* species is reported to affect immuno-physiological development, with a heightened risk for disease associated with fewer bifidobacteria and more clostridia.^27^,^28^

The composition of the enteric microbiota of infants is strongly influenced by diet. Several studies have reported that bifidobacteria and other lactic acid bacteria (LAB) dominate the microbiota of breast-fed infants, while formula-feeding generally results in a more diverse microbial population, including bifidobacteria, *Bacteroides*, clostridia and streptococci and higher numbers of facultative anaerobic bacteria, such as staphylococci, streptococci and *Enterobacteriaceae.*^1^–^3^,^29^,^30^ However, some recent studies have demonstrated that bifidobacteria only occur in a small fraction of breast-fed infants or are not numerically dominant^4^,^31^ and that coagulase-negative staphylococci are the predominant species in breast-fed infants.^32^ In other studies, different species of bifidobacteria have been shown to appear as early as four days of age in full-term breast-fed infants, and become the predominant microorganism by day six,^33^–^35^ exceeding enterobacteria by a ratio of 1000:1.^36^ Furthermore, breast-fed infants generally harbor fewer species that are liable to be pathogenic, such as *E. coli*, *C. difficile* and species of the *B. fragilis*-group.^3^ While formula-fed infants are also known to harbor bifidobacteria in the GIT, the numbers are reported to be lower than in breast-fed infants of the same age,^37^ in some studies as low as one-tenth of that encountered in breast-fed infants.^36^,^38^ Moreover, the incidence of *C. difficile* is reportedly higher in formula-fed infants compared with breast-fed infants.^38^–^40^

The dominance of bifidobacteria in breast-fed infants is attributable to the composition of human milk, which is rich in bifidogenic factors, such as oligosaccarides (i.e. lacto-N-tetraose and lacto-N-neotetraose).^41^ Oligosaccharides are carbohydrates made up of three to nine monosaccharide units^42^ and are quantitatively the third component of human milk, after lactose and lipids. A peculiar characteristic of oligosaccharides is that their monosaccharides are bound by specific bonds which are resistant to human intestinal digestive enzymes and therefore act as substrates for fermentation in the distal gut, where they promote the growth of bifidobacteria, i.e. natural prebiotics.^43^ Oligosaccharide concentration of human milk differs at different stage of lactation, with the highest concentration found in early lactation. For example, on day four of lactation, human milk contained 2 g/100 ml oligosaccharides, which declined 20% and 40% by 30 and 120 days of lactation, respectively.^44^ Breast milk is also a source of bacteria and contains up to 10^9^ microbes/L in healthy mothers.^45^ The most frequently encountered bacterial groups include staphylococci, streptococci, corynebacteria, lactobacilli, micrococci, propioni-bacteria and bifidobacteria. These bacteria originate from the nipple and surrounding skin as well as the milk ducts in the breast.^46^,^47^ Moreover, it has been demonstrated that human breast milk is a significant source of lactobacilli and bifidobacteria for the infant GIT.^48^–^50^ Human breast milk is the preferred choice for infant nutrition^51^ and numerous beneficial effects of breast milk have been demonstrated for both term and preterm infants, including neurobehavioral and cognitive development^52^–^56^ and decreased rates of infection.^57^–^59^

In contrast to human milk, oligosaccharides are virtually absent from bovine milk and thus, cows milk-based infant formula. This has led to modification of infant formula using different oligosaccharides (prebiotics) in order to improve the gut microbiota composition, to more closely resemble that obtained via breast-feeding. Prebiotics are defined as “selectively fermented ingredients that allow specific changes, both in the composition and/or activity in the gastrointestinal microbiota and that confer benefits on host well-being and health.”^60^ Thus, the role of prebiotics is to selectively stimulate the growth and/or activity of bifidobacteria and lactobacilli in the GIT. However, for a food ingredient to be classified as prebiotic, it must neither be hydrolyzed nor absorbed in the GIT, be a selective substrate for one or a few beneficial bacteria in the colon and consequently be able to alter the enteric microbiota towards a healthier composition.^17^ Oligosaccharides that have been used as prebiotics in infant formula include fructo-oligosaccharides (FOS), inulin, gluco-oligosaccharides, galacto-oligosaccharides (GOS), isomalto-oligosaccharides and xylo-oligosaccharides.^61^ Indeed, numerous studies have demonstrated that ingestion of infant formula containing prebiotics results in increased numbers of bifidobacteria and lactobacilli, over formula without prebiotics, and also decreased numbers of *E. coli*, enterococci and clostridia.^62^–^65^ Moreover, prebiotics have been demonstrated to alter the development of the immune system in infants.^66^,^67^ Moro et al.^67^ demonstrated a beneficial effect of prebiotics on the development of atopic dermatitis in a high-risk population of infants. Following supplementation of infant formula with 0.8 g/100 ml GOS/FOS (90% GOS and 10% FOS) for six months, the numbers of infants that developed atopic dermatitis was only 9.8%, compared with 23.1% of infants in the control group. In addition, infants receiving the prebiotic supplement harbored higher numbers of fecal bifidobacteria compared with the control group.^67^

The developmental aspect of the intestinal bacterial colonization of preterm infants (infants born before 37 weeks of gestation) is reported to differ from that of full-term infants. Colonization of beneficial bacteria such as lactobacilli and bifidobacteria is often delayed in preterm infants and these are only found in low numbers during the first few weeks of life, whereas colonization of potentially pathogenic bacteria such as *E. coli*, clostridia and staphylococci occurs such that these are found in high numbers.^68^–^71^ Schwiertz et al.^72^ studied the establishment of the enteric microbiota in the first few weeks of life of preterm infants by analyzing the 16S rRNA diversity in fecal samples using PCR-denaturing gradient gel electrophoresis (PCR-DGGE). Twenty nine preterm infants, hospitalized in a neonatal intensive care unit and fifteen breast-fed, full-term infants were included in the study. *E. coli*, *Enterococcus* sp. and *Klebsiella pneumoniae* were most commonly found in the fecal samples of all preterm infants, whereas for breast-fed full-term infants, bifidobacteria comprised the majority of the species present. Furthermore, in contrast to preterm infants, the genetic profiles were more diverse in fecal samples of full-term infants, indicative of a higher diversity of the bacterial community. The profiles of the preterm infants became more similar to each other over four weeks (the similarity values increased from 0% to 80% in the preterm infants compared to 18.1% to 57.4% in the full-term infants), indicating that all preterm infants harbored a similar bacterial composition, regardless of birth weight, feeding regime, and antibiotic therapy.^72^ A Japanese study reported that gut colonization in breast-fed preterm infants was characterized by high initial numbers of enterobacteria and streptococci, while bifidobacteria appeared late, at 11 days of age, and became predominant only at 19 days of age, in contrast to full-term infants who were colonized at four days of age.^70^

As preterm infants often require intensive care treatment with an increased risk of serious infections, insight in the development of the intestinal colonization of these infants is important, especially since it is hypothesized that an inappropriate colonization of the premature intestine may play a role in the development of necrotizing enterocolitis (NEC).^73^ Since preterm infants generally experience intensive care treatment and are often treated with broad spectrum antibiotics in the first days of life, this could influence intestinal colonization. Antibiotic administration results in suppression of all anaerobic bacteria, with the exception of clostridia, which remain at detectable levels, and increased numbers of *Klebsiella*, *Enterobacter*, *Citrobacter* and *Pseudomonas.*^74^,^75^ Lactobacilli and bifidobacteria are generally absent in the intestine of antibiotic-treated infants.^3^,^71^,^75^–^77^ Moreover, nursing of preterm infants in closed incubators and reduced exposure to maternal microbiota may affect the development and the diversity of their intestinal microbiota.

## Overview of the Adult Human Gut Microbiota

Once established, the human GIT is home to >100,000 billion (10^14^) bacteria, comprising over 1000 different species.^14^ Since bacteria encounter a variety of environmental conditions within the different areas of the GIT, it is not surprising that their distribution throughout the intestine varies in both concentration and population diversity (Table 1). Factors such as pH, peristalsis, redox potential, bacterial adhesion, mucin secretion, nutrient availability, diet and bacterial antagonism are all believed to influence colonization patterns.^78^

The small intestine is interposed between the sparsely populated stomach and the densely colonized bacterial microbiota of the colon. A limited number of ingested bacteria survive transit through the acidic conditions of the stomach and reach the small intestine in viable form. The lumen of the small intestine is characterized by a pH ∼7, the presence of bile salts and pancreatic secretions, which contain digestive enzymes that are themselves bactericidal, and is subjected to frequent peristaltic transit waves.^79^ Thus, the numbers of bacteria in the proximal intestine (duodenum) remain relatively low (10^4^–10^6^ bacteria/g or mL content).^80^ Acid-tolerant lactobacilli, streptococci and enterococci predominate in the upper small intestine. In contrast, the distal small intestine (ileum) accommodates a more diverse and dense microbiota (10^8^ bacteria/g or mL content). The bacterial species found in the distal small intestine include an increasing proportion of anaerobic species such as, *Bacteroides* sp., *Bifidobacterium* sp., *Enterobacteriaceae*, *Enterococcus* sp., *Streptococcus* sp., and *Lactobacillus* sp.^78^ The large intestine is a cardinal site of microbial colonization by large numbers of bacteria (10^11^–10^12^ bacteria/g or mL content) and is characterized by slow turnover, low redox potential and relatively high SCFA concentrations.^78^ The high numbers of microbes in the colon is reflected in the large proportion of fecal mass that consists of bacteria, i.e. around 60% of fecal solids.^81^ The quantitatively predominant bacteria in the human colon are members of the genus *Bacteroides, Bifidobacterium, Eubacteria, Clostridium, Lactobacillus* and gram-positive cocci.^82^,^83^ Every individual has several hundreds of microbial species, with a particular combination of predominant species that is distinct from other individuals.^84^ In contrast to the infant microbiota which is variable and dynamic in its composition over time,^85^ the GIT of an adult appears to have a microbial imprint that remains stable on a time-scale of months.^86^,^87^

## Functions of the Enteric Microbiota

Several hundred grams of bacteria living within the colonic lumen affect host homeostasis. Some of these bacteria are potential pathogens and can be a source of infection and inflammation under some circumstances, while the majority co-exist with the host and may contribute to health benefits. Examples of potentially pathogenic bacteria are staphylococci, clostridia, enterobacteria, enterococci, streptococci and *Bacteroides.*^88^,^89^ In contrast, Lactobacillus and *Bifidobacterium* species are considered among the beneficial bacteria of the GIT.^61^,^90^ Enteric bacteria confer many benefits to intestinal physiology including structural, protective and metabolic functions.^7^ Much of our understanding of the molecular mechanisms that can explain the host-bacterial mutualism comes from studies of *Bacteroides thetaiotaomicron*, a prominent member of the intestinal microbiota of humans that modulates a number of essential host functions.^91^

Along the epithelium, enteric bacteria complement the natural defense barrier against exogenous microbes, thereby preventing invasion by pathogens. Several mechanisms have been proposed for this barrier effect including displacement, competition for nutrients and epithelial binding sites, and production of antimicrobial factors such as lactic acid and bacteriocins.^92^,^93^ The microbiota is not metabolically inert, having a metabolic activity akin to that of a virtual, or hidden, inner organ.^15^,^94^ Gene diversity in this microbial community provides various enzymes and biochemical pathways that are distinct from the constitutive resources of the host. For example, SCFA such as acetate, butyrate and propionate are produced following fermentation of non-digestible prebiotic substances by certain anaerobic bacteria.^17^,^82^,^95^ SCFA in general enhance the growth of lactobacilli and bifidobacteria and play a central role in the physiology and metabolism of the colon.^95^ In addition, some of the SCFAs produced have been demonstrated to reduce the risk of developing diseases, such as colon cancer and inflammatory bowel disease (IBD).^11^,^96^ Resident bacteria can also metabolize dietary carcinogens, synthesize vitamins such as biotin, folate and vitamin K, and assist in the absorption of calcium, magnesium and iron.^82^,^97^–^99^ Overall, the benefits of this complex metabolic activity are recovery of metabolic energy and absorbable substrates for the host, and supply of energy and nutritive compounds for bacterial growth and proliferation. It has also been proposed that the gut microbiota of individuals has a specific metabolic efficiency, and differences in microbial composition between individuals might regulate energy storage and predispose to obesity.^100^,^101^ Moreover, the enteric microbiota is a metabolically active partner in host defense that influences the normal structural and functional development of the mucosal immune system. Establishment of a normal microbiota provides the host with a substantial antigen challenge, with a strong stimulatory effect for maturation of the gut associated lymphoid tissue (GALT) and mucosal immunity.^102^,^103^ The fact that approximately 80% of all immunologically active cells of the body are located in the GALT is an affirmation of the importance of microbe-gut immune system interactions.^104^ Indeed, studies have shown that germ-free mice have an under-developed sparse mucosal immune system, with small Peyer’s patches without germinal centers and small T cell zones. Furthermore, their lamina propria contains essentially no immunoglobulin A (IgA), plasma cells or CD4 cells, and intraepithelial lymphocytes are also rare compared with conventional animals.^92^,^105^ However, reconstitution of germ-free mice with an intestinal microbiota leads to a rapid expansion of the immune system.^106^ Intestinal bacteria are not uniform in their ability to drive mucosal inflammatory responses. Some species such as *Bacteroides vulgatus* are proinflammatory,^107^ while other species such as bifidobacteria and lactobacilli lack inflammatory capacity.^15^,^108^ The ability of immunosensory cells, such as enterocytes, M cells, and dendritic cells to discriminate pathogenic bacteria from commensal bacteria is mediated in part, by two major host pattern recognition receptor (PRR) systems—the family of Toll-like receptors (TLRs) and the nucleotide-binding oligomerization domain/caspase recruitment domain isoforms (NOD/CARD).^109^ These PRRs have a fundamental role in immune-cell activation in response to specific microbial-associated molecular patterns such as lipopolysaccharide (LPS), lipotechoic acid, peptidoglycan and flagellin. Many PRR ligands are expressed by commensal bacteria, nonetheless the healthy gut does not evoke inflammatory responses to these bacteria. Conversely, some commensal bacteria such as bifidobacteria and lactobacilli exert protective effects by attenuating proinflammatory responses induced by different pathogens.^108^,^110^ Recent evidence is also emerging to show that certain enteric bacterial components can ameliorate radiation induced mucosal injury.^111^,^112^ Thus, it is possible that the composition of the enteric microbiota influences individual variations in immunity.

## Evidence for Probiotic Treatment in the Management of Common Infant Diseases Associated with the Gut Microbiota

**Figure 1:**
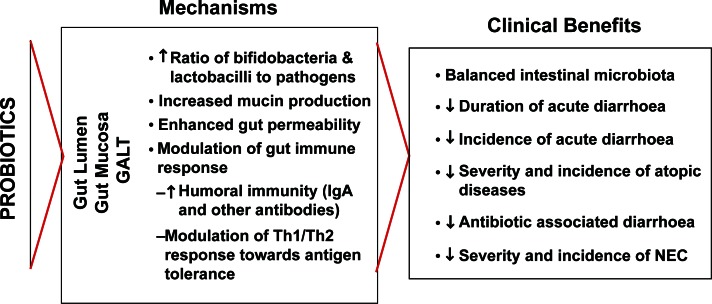
Summary of reported mechanisms and related clinical benefits of probiotics in pediatrics.^117^

Probiotics are defined as “live microorganisms which, when administered in adequate amounts, confer a health benefit on the host.”^113^ The bacteria most commonly used as probiotics belong to the genera *Lactobacillus* and *Bifidobacterium*. Manipulation of the microbiota using probiotics in infants has shown promising results in the prevention and treatment of diseases such as diarrhea, allergy and NEC (Fig. 1).^114^–^118^ The precise mechanisms behind these health-promoting effects are not fully understood, but include normalization of microbiota, reduction in intestinal permeability, increase in mucosal barrier function, protection against invasion by pathogens, production of beneficial metabolites and anti microbial substances and stimulation of immunity^117^ (Fig. 1).

By far the best-studied clinical outcome with the use of probiotic bacteria in children has been that of treatment of acute infantile diarrhea.^117^ Acute diarrhea is a serious cause of infant morbidity and mortality caused by a range of different factors. Bacterial infections caused by *Shigella*, *Salmonella* and *Campylobacter*, viral gastrointestinal infections (mainly rotavirus) and antibiotic treatment have all been associated with acute diarrhea in infants.^119^ Oral administration of probiotics have shown benefits in infantile diarrhea in a number of studies, including decreased frequency of infections, reduction in the severity and length of the diarrhea episode, decreased shedding of rotavirus and promotion of systemic and local immune responses.^116^,^120^ For example, *L. rhamnosus* GG has repeatedly been shown to reduce the duration of infant diarrhea by about 50%,^121^ while Saavedra et al.^119^ reported that administration of *Bifidobacterium bifidum* and *Streptococcus thermophilus* to infants reduced the incidence of diarrhea four-fold compared with unsupplemented controls. Moreover, Correa et al.^122^ demonstrated that supplementation with *Bifidobacterium lactis* and *S. thermophilus* to infants resulted in a 50% reduction of antibiotic-associated diarrhea compared with controls.

NEC is the most common serious, acquired gastrointestinal disease in the preterm infant, which is characterized by impaired mucosal barrier function and increased gut permeability. Although many variables are associated with NEC, only prematurity has been consistently identified in case-controlled studies.^123^ In infants weighing less than 1,500 g at birth, there is a 10% incidence of NEC, with mortality rates ranging from 25% to 30%.^124^ Several bacterial species have been associated with NEC, including members of *Enterobacteriaceae*, *Clostridia*, and coagulase-negative staphylococci.^125^–^127^ A number of reports suggest that probiotics may play a role in the control or prevention of NEC in preterm infants. A recent Cochrane review by Alfaleh and Bassler^128^ compared the efficacy and safety of prophylactic enteral probiotic administration versus placebo or no treatment in the prevention of severe NEC in preterm infants. Nine eligible trials randomizing 1,425 infants were included. However, included trials were highly variable with regard to enrolment criteria (i.e. birth weight, and gestational age), baseline risk of NEC in the control groups, timing, dose, formulation of the probiotics used and feeding regimes. It was concluded that enteral supplementation of probiotics can reduce the risk of severe NEC and mortality in preterm infants.^128^ Moreover, Lin et al.^129^ reported that probiotic administration reduced the incidence of NEC by >50% when *L. acidophilus* and *B. infantis* were administered to infants weighing <1,500 g. Bin-Nun et al.^130^ demonstrated that administration of a probiotic mixture (*B. infantis*, *S. thermophilus* and *B. bifidus*) to infants weighing ≤1,500 g reduced the incidence of NEC by about 25%. Possible mechanisms by which probiotics may protect against onset of NEC include prevention of bacterial migration across the mucosa, competitive exclusion of pathogenic bacteria and enhancement of immune responses.^128^,^131^,^132^

Research to date supports the importance of the early human intestinal microbiota on the development of allergic diseases such as atopic eczema, asthma and food allergy. Bacterial colonization of the GIT after birth is essential to redress the balance of the skewed T-helper-cell type 2 immune response present in the newborn infant. This normal interaction between infant and microbes is thought to be compromised in the Western world, with a reduction in bifidobacteria and an increase in clostridial species, particularly in formula-fed infants.^133^ Differences in intestinal microbiota have been described between healthy children and those exhibiting allergic diseases.^134^,^135^ In a prospective study, children who later developed allergic sensitization to common allergens were shown to have lower numbers of fecal bifidobacteria and increased numbers of clostridia from the first weeks of life.^27^ Bifidobacteria have been associated with a lower risk of atopy.^27^,^135^,^136^ Sudo et al.^137^ reported that oral tolerance was achieved in germ-free mice only if the intestinal microbiota was reconstituted with bifidobacteria during the infant period. Components of the potentially pathogenic microbiota such as LPS have been reported to be involved in the development of atopic eczema.^27^,^138^ Probiotics have been reported to help prevent and/or manage atopic diseases and allergies in infants. Isolauri et al.^114^ demonstrated that supplementation of infants with atopic eczema with *Bifidobacterium animalis* subsp*. lactis* Bb12 or *L. rhamnosus* GG resulted in earlier recovery than standard treatment after two months. Similar findings were reported for *Lactobacillus fermentum* VRI-003, which led to an improvement in the extent and severity of atopic eczema, when administered to infants for eight weeks.^139^ In addition to treatment of allergy, it has been reported that probiotics can also reduce the risk for developing the disease. In this respect, one of the earliest studies was performed with a non-pathogenic *E. coli* strain which was administered to term and preterm infants. At 10 and 20 years of follow-up, subjects treated with the *E. coli* strain during infancy experienced significantly fewer allergic diseases than untreated controls.^140^ This study demonstrated that it is possible to direct the immune system towards tolerance in infants in which the immune system is still immature.

## Conclusions

The role of the enteric microbiota is undoubtedly an important factor governing infant health and probably has an effect on overall health status in later life. Indeed, the ‘fetal programming hypothesis’ as proposed by Barker, suggests that disturbed intrauterine growth has a negative influence on the development of the cardiovascular system and favors the occurrence of hypertension, insulin resistance, hypercholesterolemia, and hyperuricemia in adult life.^141^ Thus, influencing the composition of the gut microbiota in early life may impact on tendency towards the development of certain diseases in later life. Several factors may promote a greater microbial diversity in infants, such as breast milk feeding, vaginal delivery and avoiding antibiotics, which could contribute to enhanced infant health. Moreover, the use of pre- and probiotics may play an important role in preventative health and in the management of specific conditions in infants by increasing the numbers of lactobacilli and bifidobacteria in the intestine. Groups who may benefit from such interventions include formula-fed infants, infants born by Caesarean section, premature infants, and infants treated with antibiotics. However, current evidence justifying such interventions is limited and adequately powered studies addressing these issues are keenly awaited. In particular, further large randomized controlled trials are required to investigate the potential benefits and safety profile of probiotic supplementation in extremely low birth weight infants (ELBW) for the prevention of NEC.

## Figures and Tables

**Table 1:** Bacteria within different regions of the adult human GIT.^80^

**Predominant genera of bacteria (colony forming units/mL or/g)**		

**Stomach and duodenum**	**Jejunum and ileum**	**Colon**
10^1^–10^3^	10^4^–10^8^	10^10^–10^12^
Lactobacilli	Lactobacilli	*Bacteroides*
Streptococci	Enterobacteria	Bifidobacteria
Yeast	Streptococci	Streptococci
	*Bacteroides*	Fusobacteria
	Bifidobacteria	Enterobacteria
	Fusobacteria	Clostridia
		*Veilonella*
		Lactobacilli
		Proteus
		Staphylococci
		*Pseudomonas*
		Yeast
		Protozoa
